# Limited inbreeding avoidance at the gamete level despite inbreeding depression in Atlantic salmon (*Salmo salar*)

**DOI:** 10.1111/1365-2656.70123

**Published:** 2025-09-03

**Authors:** Marco Graziano, Monica Solberg, Kevin A. Glover, Martin Taylor, Anne Grete Eide Sørvik, David Murray, Simone Immler, Matthew J. G. Gage

**Affiliations:** ^1^ Centre for Ecology, Evolution, and Conservation, School of Biological Sciences University of East Anglia Norwich UK; ^2^ Department of Comparative Biomedicine and Food Science University of Padova Legnaro Italy; ^3^ Population Genetics Group, Institute of Marine Research Bergen Norway; ^4^ Centre for Environment Fisheries and Aquaculture Science (CEFAS) Lowestoft UK

**Keywords:** cryptic female choice, fertilisation, inbreeding, kin recognition, ovarian fluid, sperm competition

## Abstract

1. Inbreeding and the associated increase in homozygosity and potential accumulation of deleterious alleles may reduce fitness in a process known as inbreeding depression. Mechanisms to mitigate reproduction between close relatives, ranging from pre‐mating mate choice to post‐mating gamete selection, have evolved across taxa. In external fertilisers like Atlantic salmon (*Salmo salar*), where females have limited control over paternity, mechanisms of inbreeding avoidance can be expected to evolve at the gamete level.
2. Philopatric Atlantic salmon may run the risk of breeding between relatives, particularly in small populations, and frequent escapes from aquaculture settings are augmenting the chances of adults from the same sibling cohort overlapping in the wild, raising potential ecological and sustainability concerns. Moreover, the presence of inbreeding avoidance mechanisms between full siblings in externally fertilising fish is currently untested.
3. This study tested post‐mating inbreeding avoidance mechanisms in domesticated Atlantic salmon. In a paired breeding design, we compared sperm motility parameters in sibling and non‐sibling ovarian fluid, and assessed fertilisation and hatching success, growth rate and paternity in sperm competition trials between sibling and non‐sibling males.
4. Sperm activated in ovarian fluid of sibling females showed lower values of motility‐related parameters and led to an average of 18% reduction in fertilisation rates in the resulting crosses. Furthermore, offspring from sibling crosses were smaller before the onset of sexual maturation. However, we found no difference in survival rates between sibling and non‐sibling cross offspring. Besides, when sperm from sibling and non‐sibling males were competing simultaneously for the same egg batch, we found no influence of this on paternity.
5. Our findings reveal the presence of post‐mating inbreeding avoidance at the gamete level in Atlantic salmon, but its effects are limited in competitive scenarios.
6. Our results have implications for salmonid conservation and aquaculture, where small natural or closed domestic strains may both display a degree of inbreeding. Mating between escaped domestic and wild salmonids could favour admixed over wild or feral crosses if an inbreeding avoidance mechanism is present; although this remains to be tested in more outbred crosses.

## Graphical abstract

Our study investigates inbreeding avoidance mechanisms at the gamete level in domesticated Atlantic salmon using sperm motility traits assessments, fertilisation trials, and paternity analysis under competitive conditions. We demonstrate that ovarian fluid from sibling females reduces sperm motility traits and fertilisation rates but found no significant differences in offspring survival or paternity outcomes under sperm competition. These findings provide critical insights into the limited efficacy of post‐mating mechanisms for inbreeding avoidance in domesticated salmon, highlighting implications for aquaculture management and wild salmonid conservation. Moreover, the presence of inbreeding avoidance mechanisms between full siblings in externally fertilising fish is currently untested.

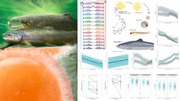

## 1 INTRODUCTION

Inbreeding and the resulting reduced genetic variation can lead to detrimental effects known as inbreeding depression (Charlesworth & Charlesworth, 1987) including reduced adaptive responses, reproductive success and survival, which increase the risk of population extinction (Caughley, 1994; Hedrick, 1999, 2000). Inbreeding and inbreeding depression have been documented in the wild and particularly affect the viability of small populations (Bijlsma et al., 2000; Keller & Waller, 2002) presenting a primary concern in conservation biology (Hedrick, 2000).

From a theoretical perspective, inbreeding avoidance is expected to evolve when inbreeding depression is substantial and the likelihood of mating with relatives is non‐negligible. However, theory also predicts that under conditions of intermediate inbreeding depression and low opportunity costs for males, selection may favour inbreeding in males but avoidance in females (Parker, 2006), giving rise to sexual conflict over inbreeding and complicating predictions about mating outcomes.

Recent meta‐analytical studies (de Boer et al., 2021; Pike et al., 2021) have brought important nuance to these expectations. Pike et al. (2021) have shown that mate choice based on kinship occurs exclusively when there is a concrete risk of encounters between related mates and when there is inbreeding depression. Otherwise, kinship‐based mate choice will be weak, even in the presence of a costly inbreeding depression. The work from de Boer et al. (2021) has importantly shown that mating with kin is rarely avoided, also evidencing a publication bias towards studies showing kin avoidance, overturning the widely held view that animals tend to avoid inbreeding.

In the cases where kin discrimination or mate choice for genetic compatibility does occur, one influential idea is the ‘good genes as heterozygosity’ hypothesis. This hypothesis implies that females assess the genetic quality of potential mates by evaluating genetic compatibility with their own genotype, thereby promoting heterozygosity in offspring (Landry et al., 2001; Qvarnström & Forsgren, 1998). Although the concept of ‘mate quality’ itself is difficult to define, and the genetic basis for incompatibility between mates is likely multifactorial (Tregenza & Wedell, 2000; Zeh & Zeh, 1996, 2001), several studies support the potential for such compatibility‐based mate choice mechanisms.

A growing body of studies has investigated the various strategies by which organisms could avoid inbreeding, including differential dispersal of one or both sexes, kin recognition and mate choice (Olsen et al., 2020; Pusey & Wolf, 1996; Szulkin et al., 2013). Inbreeding avoidance mechanisms have evolved to control the rate of mating between close relatives before, during and after mating. At the premating stages, sex‐biased dispersal or kin recognition tactics, for instance, are evolutionary routes to reduce the probability of inbreeding (Pusey & Wolf, 1996; Tregenza & Wedell, 2000). At the post‐mating stage, cryptic female choice and preference for sperm from non‐related males, as well as reduced parental investment in offspring sired between relatives, have been reported (Simmons, 2005; Zeh & Zeh, 1996, 1997). In vertebrates, the main supporting evidence of inbreeding avoidance mechanisms resulted from studies on kin recognition based on major histocompatibility complex (MHC), which seems to play a role in mate selection based on genetic compatibility (Keane, 1990; Landry et al., 2001; Simmons, 1991). However, the mechanistic cascade of events that enables any mate choice has not yet been identified, stimulating the proposal of different explanatory theories (Ziegler et al., 2005).

Females are thought to be able to influence paternity at different stages of the reproductive process, before, during and after mating, and before and after fertilisation (Birkhead, 1998b; Birkhead et al., 1993; Gowaty, 1994; Zeh & Zeh, 1997). Several studies support female control of paternity before copulation (Gowaty, 1994). Female butterflies (*Bicyclus anynana*) (Fischer et al., 2015) and female sticklebacks (*Gasterosteus aculeatus*) (Frommen & Bakker, 2006), for example, show pre‐mating preferences towards unrelated mates. On the other hand, post‐copulatory inbreeding avoidance has been reported in red junglefowl (*Gallus gallus*) (Pizzari et al., 2004) and the cricket species *Teleogryllus oceanicus* (Simmons et al., 2006) and *Gryllus bimaculatus* (Bretman et al., 2009). However, pre‐ and post‐mating mechanisms are not mutually exclusive, and their co‐occurrence has been shown across different species such as the Trinidadian guppies (*Poecilia reticulata*) (Daniel & Rodd, 2016; Gasparini & Pilastro, 2011) and house mice (*Mus domesticus*) (Firman & Simmons, 2008; Penn & Potts, 1998). Besides, disentangling the role of the two sexes in determining reproductive success is challenging (Birkhead, 1998a). Externally fertilising organisms are particularly well suited to overcome these challenges because they offer an opportunity to study gamete interactions in a controlled yet natural fertilisation microenvironment. Moreover, understanding whether and how inbreeding avoidance occurs in externally fertilising species is not only relevant for better characterising their reproductive biology, but also for identifying whether and under what conditions external fertilisation can promote the evolution of post‐copulatory inbreeding avoidance—a possibility that may seem counterintuitive given the presumed lower opportunity for selective mate choice in these systems. Still, some of the clearest demonstrations of sperm discrimination have been recorded in externally fertilising organisms (such as the fishes *Symphodus ocellatus* (Alonzo et al., 2016), Atlantic salmon (*Salmo salar*) (Yeates et al., 2009) and the blue mussel *Mytilus galloprovincialis* (Evans & Lymbery, 2020; Oliver & Evans, 2014)). Notably, the meta‐analysis from de Boer et al. (2021) has shown that externally fertilising organisms constitute only a very minor portion (2.7%) of the studies on inbreeding avoidance. Therefore, understanding if and how these organisms practice inbreeding avoidance, and at which degree, could be of great interest to shed light on this underrepresented reproductive mode.

Atlantic salmon (*Salmo salar*) are anadromous fish distributed across rivers on both sides of the North Atlantic. Juveniles spend several years in freshwater before migrating to oceanic feeding grounds, where they remain for 1–3 years before returning to their natal streams to spawn (Garant et al., 2000; Jordan & Youngson, 1992). Reproduction, characterised by external fertilisation and opportunities for competitive mate choice, occurs in rivers during autumn and early winter (Fleming, 1996). In natural populations, although juvenile survival to adulthood varies, many return to natal streams, potentially overlapping with siblings (Davidsen et al., 2013; Thorstad et al., 2008, 2011). Kin recognition and sibling interactions are documented among juvenile Atlantic salmon (Brown, 1997; Brown & Brown, 1996; Dittman & Quinn, 1996; Nordeng, 1977). For instance, siblings that were reared separately were found to shoal together in experimental streams (Olsén et al., 2004). Wild adult fish captured at sea showed instead no evidence of shoaling between full siblings (Palm et al., 2008), and while the homing behaviour of this species is documented (Davidsen et al., 2013; Hansen et al., 1987, 1993), it remains unclear whether wild adults from a sibling cohort temporally and spatially overlap on spawning grounds. Conversely, recapture programmes have revealed the occurrence of hatchery‐bred adult kin groups comprised of full‐sibling families in the same river where they were introduced (Herbinger et al., 2006).

In the past three or four decades, many wild salmon populations have been subjected to introgression from domesticated farmed escapees in regions where aquaculture and wild populations co‐exist, raising concerns over the evolutionary consequences of this (Glover, Solberg, et al., 2017). One of these concerns is linked to the fact that domesticated salmon strains have finite population sizes, and thus display reduced genetic variation in comparison with wild conspecifics (Karlsson et al., 2010, 2011; Skaala et al., 2004, 2005). Despite ongoing debate over the straying rates of farmed salmon crosses (e.g. Diserud et al., 2022; Jonsson & Jonsson, 2017; Skaala et al., 2019), kinship analyses among farm‐escaped salmon in the wild have detected small numbers of adult full‐siblings (Madhun et al., 2017; Quintela et al., 2016), indicating that opportunities for sibling mating, while limited, do exist. Although introgression of domesticated salmon in wild populations may temporarily lead to increased genetic variation (increased allelic richness) in the wild recipient population due to transference of novel genotypes (Skaala et al., 2006), it erodes genetic differentiation among wild populations (Glover et al., 2012). Mating between genetically divergent escaped domestic and wild fish could thus favour domesticated × wild, or admixed × wild crosses over wild × wild (or domesticated × domesticated) crosses if an inbreeding avoidance mechanism is present. Nevertheless, paternity screenings in the wild have revealed surprisingly high levels of multiple paternity, with eggs from a single nest being fertilised by up to 16 different males, including large anadromous males as well as small mature male parr (Weir et al., 2010). It could therefore also be debated that if a strong inbreeding avoidance mechanism is present, this could further speed up the introgression by not only favouring non‐related domesticated males but also admixed or feral mature male parr (Holborn et al., 2022).

It has been argued that non‐random gamete fusion may not evolve because the costs may be high in external fertilisers, where the rapid fusion of gametes may reduce the chance for assortative mating (Wedekind et al., 2004). Nevertheless, in salmonids as well as other fish, evidence for cryptic female choice mediated by the ovarian fluid, a viscous substance released with eggs at oviposition, is strong (Graziano et al., 2023; Rosengrave et al., 2016; Yeates et al., 2013), as well as there being evidence for non‐random mating choice, at least at the diploid level (Alonzo et al., 2016; Landry et al., 2001) as opposed to the haploid one (Promerová et al., 2017). However, the presence of inbreeding avoidance mechanisms between full siblings in externally fertilising fish is currently untested. Therefore, there is a need to assess if a discriminatory power exists at the gamete level so that the reproductive interaction between closely related individuals can be comprehended more thoroughly, finding applicability in conservation breeding programmes, in predicting the ecological interactions following escapes and to optimise fertility protocol and sustainability within the farms. The capability of creating full‐sibling and non‐sibling crosses of domestic strains using a full factorial design in which the same male can be tested as either a non‐sibling or sibling when paired to a specific female makes Atlantic salmon an excellent model to study non‐random mate choice in the context of inbreeding avoidance. Finally, testing inbreeding avoidance mechanisms at the gamete level, in the presence of reduced female control over paternity (external fertilisation), offers a perfect opportunity to study the evolution of post‐mating pre‐zygotic mechanisms of inbreeding avoidance (Jordan & Bruford, 1998).

In the present study, we tested how genetic relatedness between males and females affects sperm behaviour and sperm–egg incompatibility and studied the mechanisms of inbreeding avoidance found in domesticated Atlantic salmon. We performed in vitro fertilisation assays to analyse sperm motility and velocity parameters, compared offspring fitness between sibling and non‐sibling crosses and ran sperm competition experiments to test relative reproductive success between sibling and non‐sibling males. These assays allowed us to explore whether a potential self‐ versus non‐self‐recognition occurs strictly between gametes or also between sperm and the ovarian fluid.

## 2 MATERIALS AND METHODS

### 2.1 Experimental fish

All fish used in this experiment originate from the domesticated aquaculture strain Mowi, which is one of the earliest strains used in salmon aquaculture and has been in a closed breeding regime for >15 generations at the time of this study. Since the late 1960s, this strain has been directionally selected for growth, delayed gonadal maturation and fillet quality, among other traits. This has resulted in significant genetic divergence from wild salmon in multiple traits such as growth under farming conditions (Glover et al., 2009; Solberg, Skaala, et al., 2013) and survival in the wild (Besnier et al., 2015; Skaala et al., 2019).

**FIGURE 1:**
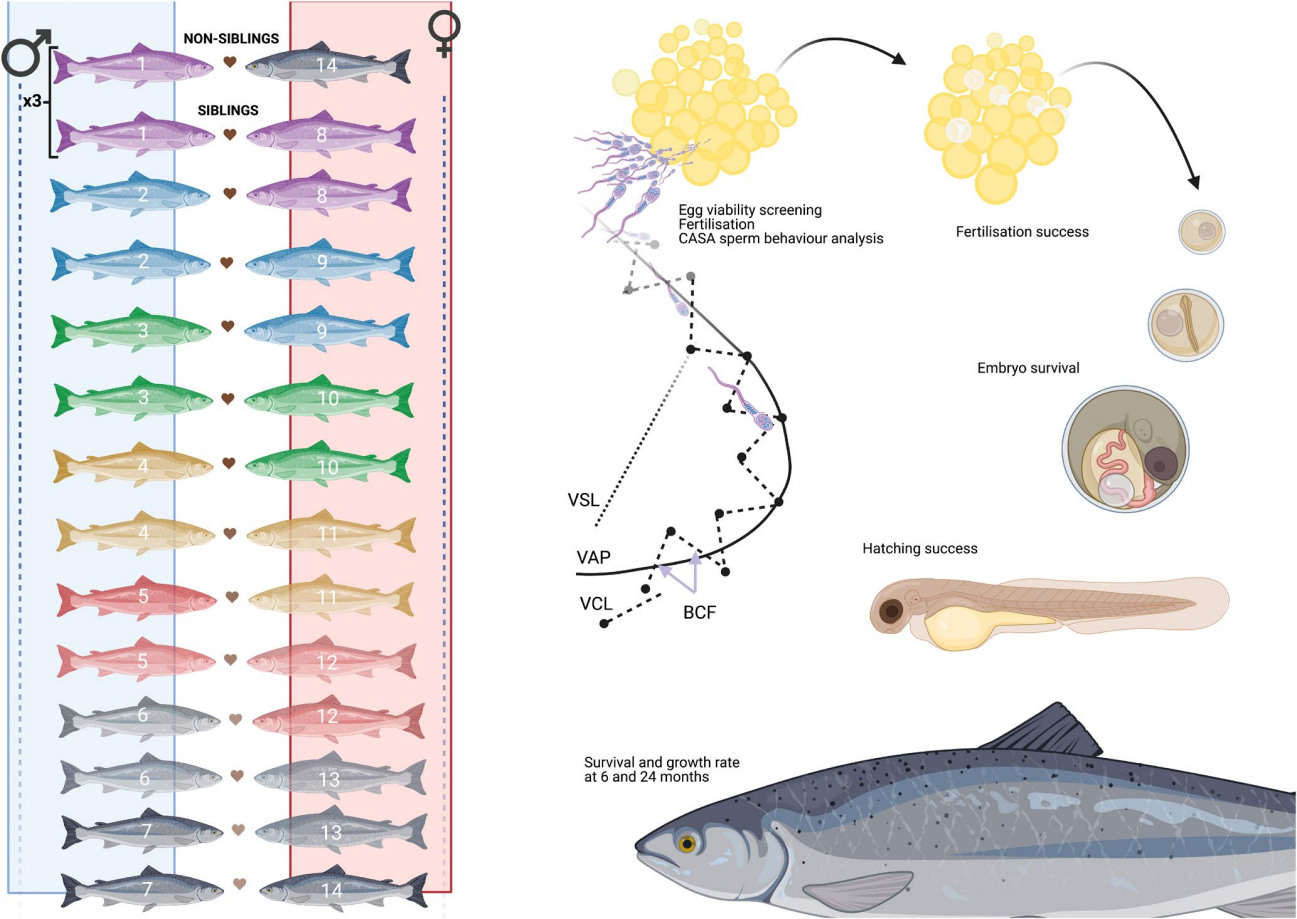
Experimental design used in the homospermic experiment for sperm swimming behaviour, fertilisation, hatching success and growth rates in non‐siblings and sibling salmon crosses (*N* = males × 7) females arranged in 28 unique crosses (3 replicates per cross). Individuals are siblings when they share the same colour.

At the Institute of Marine Research in Norway (IMR), multiple generations of domesticated and wild Atlantic salmon populations have been produced (Glover et al., 2018; Harvey et al., 2016, 2018; Perry et al., 2019; Solberg et al., 2014; Solberg, Skaala, et al., 2013). All the parental fish used in this study were from a well‐known genetic background and known pedigrees. Information about relatedness among the parental fish was provided by the commercial producer at the start of the experiment. Based upon their family/genetic background, seven males and seven females were selected to generate seven full sibling pairs and seven non‐sibling pairs, with each fish acting either as a sibling or as a non‐sibling across different pairs (see Figure 1).

### 2.2 Gamete collection

Brood fish were stripped for gametes at the Mowi breeding facility in Askøy (western Norway). Seven males and seven females, reared under controlled aquaculture conditions throughout their life cycle, were stripped of gametes. Stripping was conducted by using standard hatchery procedures as described in Gage et al. (2004). Briefly, male gametes were collected from the urogenital pore by applying gentle abdominal pressure. Prior to stripping, urogenital pores were dried with a paper towel to avoid sperm activation before the start of the experiments due to contamination with water, mucus or urine. Once collected, milt samples were transferred into sterile flasks and kept on ice. Eggs, including the ovarian fluid from the females, collected by cutting the abdominal cavity open, were placed in sealed egg buckets and kept on ice. Gametes were then transported on ice and processed on the same day at the experimental aquaculture facilities and laboratories at the IMR Research Station in Matre.

### 2.3 Sperm behaviour assessment

For each of the seven experimental males, an aliquot of 1.5 mL of ejaculate was collected from the main flask after gentle mixing to homogenise the sample, transferred into an Eppendorf vial and placed on ice. From each vial, 0.8 μL of undiluted ejaculate was directly activated in a 4‐μL solution of 100% ovarian fluid under a microscope (UOP, Tokyo, Japan, equipped with a 20× negative‐phase contrast objective) using double‐chambered Micro tool™ Cytonix sperm slides (Cytonix, Beltsville, MD 20705, USA). These slides are specifically designed to ensure quick and homogenous mixing, reduce wall effects and stop the baseline flow caused by pipetting within the shortest amount of time, to produce trustable sperm activity recordings at 5 s post activation (spa). The aliquot was placed at the entrance of the chamber and subsequently activated by flushing the ovarian fluid through the entrance to fill the chamber. Video recording started at 5 s post activation to record sperm activity through a camera (Grasshopper2 digital camera, FLIR systems®, British Columbia, Canada) mounted to the microscope. For every male, sperm activity in ovarian fluid belonging either to a non‐sibling or to a full‐sibling female was recorded in three experimental replicates per sample, from 5 to 60 spa. The resulting videos were exported and analysed in a CASA automated plug‐in available for the FIJI ImageJ software (Schindelin et al., 2012) as described in Purchase and Earle (2012). Briefly, recorded videos were converted to binary b/w images, labelled with information about male and female IDs, experimental replicate and relatedness of the cross (sibling or non‐sibling) and organised in a folder. Preliminary trials on a smaller sample of videos were performed to obtain the optimal input parameters to feed to the CASA software (details on parameters can be found in the Figure S5). The per cent of motile cells (MOT), curvilinear velocity (VCL), straight line velocity (VSL), average path velocity (VAP), linearity (LIN = VSL/VCL), beat cross frequency (BCF), wobble (WOB) and progression (PROG) were collected for both individual cells and the averages for every second calculated. All sperm motility and fertilisation trials were performed at a water temperature of 6–7°C and similar air temperature.

### 2.4 Fertilisation and hatching success

For all experimental crosses (sibling or non‐sibling), two replicates of 100 eggs each were collected to conduct fertilisation trials and to monitor development to embryo stage. An additional 500 eggs per cross were monitored for post‐hatching development. Batches of 100 eggs each were fertilised by using 100 μL raw milt added per fertilisation and activated in 100 mL natural river water at the temperature of 6 ± 0.62°C. After 2 min (maximising fertilisation potential), fertilised egg batches were transferred into separate 7 × 7 cm hatching chambers. At this stage, eggs were gently mixed within the hatching chambers, counted and screened for fertility (unfertilised eggs quickly turn white shade with patches of colour). Unfertilised eggs were counted and removed from the chamber. All the trays containing fertilised egg batches were kept in a controlled oxygen saturated flow‐through system at a temperature of 6 ± 1.7 until hatching. All the perforated PVC trays shared the same water during development and labelled groups were evenly distributed, ensuring an equal number of sibling and non‐sibling egg batches in each tray. At hatching, all embryos were counted and immediately placed in a 60% Etoh solution for subsequent analyses.

The batches of 500 eggs were fertilised using 500 μL raw milt and placed in the same flow‐through system but in 15 × 15 cm hatching chambers. During development, each batch was checked daily to monitor embryonic development, to count embryonic deaths and to remove dead eggs/embryos from the hatching chamber. After hatching, alevins were randomly divided into three equally sized groups per replicate and transferred into six (three for non‐sibling crosses and three for sibling crosses) larger tanks (1000 L) resulting in three replicates per experimental group. The alevins and juvenile fish were fed ad libitum from the start of the feeding phase. After 6 months, half the fish were collected, euthanised, and their body weight (BW) and fork lengths (FL) were measured. The remaining half was split again into two groups and transferred into bigger tanks (5 × 5 m each) and fed ad libitum for 2 years under natural photoperiod in natural seawater under the same conditions described above. After 2 years, the remaining fish were sampled for growth‐related parameters.

### 2.5 Sperm competition

**FIGURE 2:**
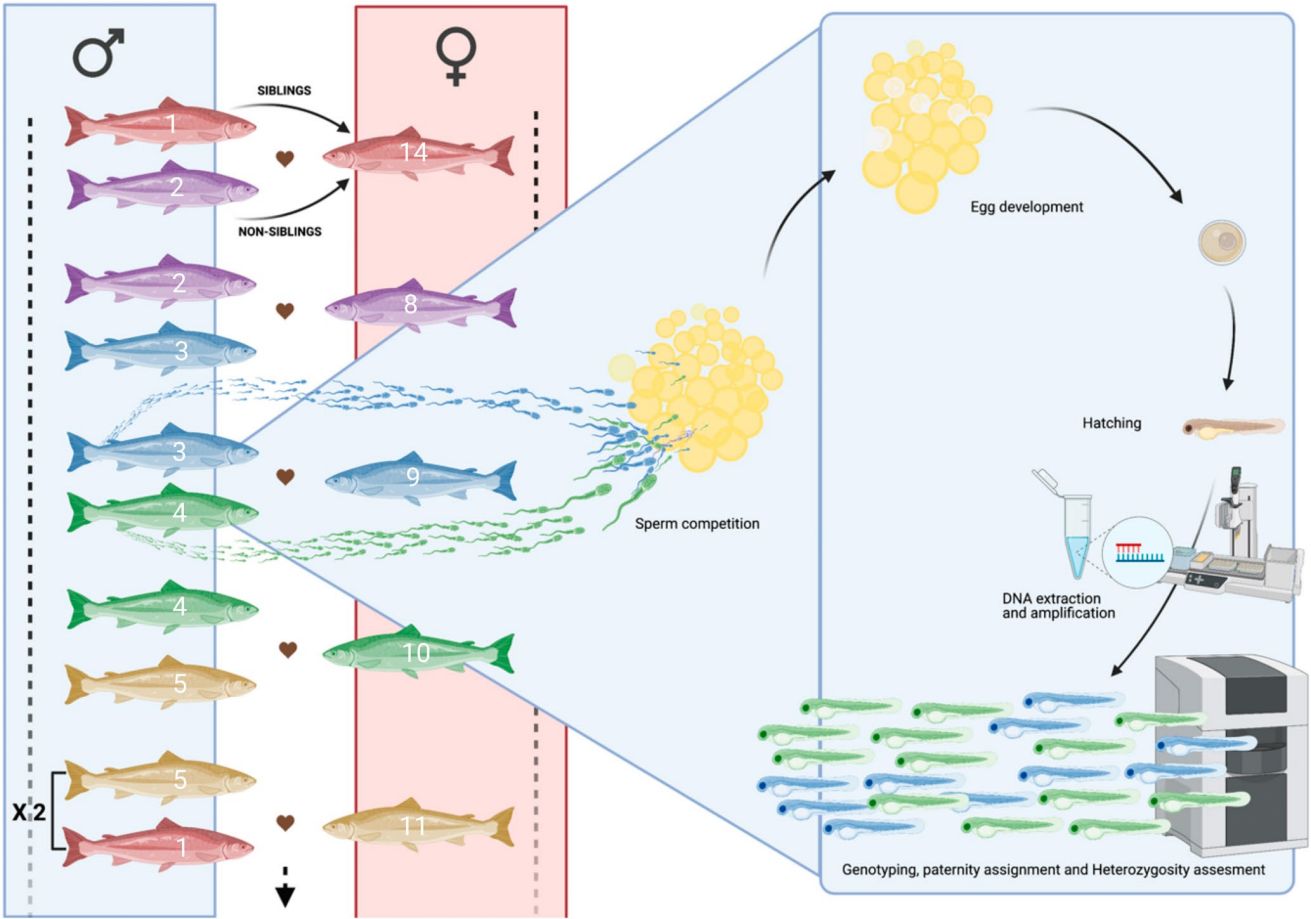
Experimental design for the sperm competition (heterospermic) experiment; (*N* = 7 males × 7 females arranged in seven unique trios (two replicates per trio)). Individuals are siblings when they share the same colour.

Approximately 100 eggs from each female were fertilised using a total of 200 μL of sperm from two males (100 μL from a full sibling male and 100 μL from a non‐sibling male). Individuals were paired according to the same study design as for the fertilisation trials described above (Figure 2). The separate sperm samples from each male were gently homogenised with a pipette, and the samples from the sibling and non‐sibling males were placed on opposite sides of a dry 1 L plastic beaker having an inner concave portion containing the eggs to avoid premature sperm activation. Sperm and eggs were simultaneously activated and mixed by the addition of 200 mL natural river water at 6 ± 0.62°C. Immediately after the addition of water, pictures of the egg batches were taken for later counting of the total number of eggs. As described above, the fertilised and hardened eggs were transferred into the flow‐through hatchery system and monitored until hatching. Hatched alevins were counted and 48 individuals per family were randomly sampled, euthanised and placed in 70% EtoH for subsequent paternity analyses.

### 2.6 Genotyping and paternity assignment

To assign parents to the offspring resulting from the sperm competition trials and examine the inheritance patterns in the hatched embryos, individuals were genotyped using a panel of five microsatellites: *SsaOsl85* (Slettan et al., 1995), *MHC1* (Grimholt et al., 2002), *MHC2* (Stet et al., 2002), *Ssa197* (O'Reilly et al., 1996) and *SsaF43* (Sánchez et al., 1996). The selected set of microsatellites has been used for more than a decade on tens of thousands of Atlantic salmon genotyped for population genetic studies (Harvey, Tang, et al., 2017), pedigree identification (Jørgensen, Solberg, et al., 2018; Solberg, Zhang, et al., 2013), to identify the source of farmed salmon escapes (Glover et al., 2015, 2016; Glover, Hansen, et al., 2017; Harvey, Fjelldal, et al., 2017; Jørgensen, Wennevik, et al., 2018).

DNA was extracted from one eye of each alevin in 96‐well format using the HotSHOT method as described by Truett et al. (2000). On each DNA extraction plate, two blank cells were added to serve as negative controls. PCRs were run for the five selected microsatellites and PCR products were analysed on an ABI 3730 Genetic Analyser (Applied Biosystems, Foster City, CA) and sized by a 500LIZä size standard. Alleles binned automatically in the programme Genemapper were manually checked by two researchers before exporting data for statistical analysis. Offspring paternity assignment was conducted adopting the exclusion‐based family assignment programme FAP (Taggart, 2006) permitting the linking of offspring to their familiar origins for known parental genotypes and crosses. The use of exclusive male + male × female in each of the crosses enabled unequivocal paternity assignment because each individual alevin could only have been sired by one of the two males in each trio.

### 2.7 Statistical analyses

All analyses were performed using R Studio version 1.3.1093 (RStudio, 2021, Integrated Development for R. Rstudio, PBC, Boston, MA) equipped with *car* (Fox & Weisberg, 2018), *glmmTMB* (Brooks et al., 2017), (*readxl*), (*lme4*), (*lmerTest*), (*DHARMa*), (*lsmeans*), (*merTools*), (*dplyr*), (*tidyverse*), (*rstatix*), (*ggpubr*), (*arsenal*), (*knitr*), (*survival*) and *lmerTest* (Kuznetsova et al., 2017) packages to perform exploratory analysis, run the main models, perform post‐hoc tests and create output tabs. Graphical figures were plotted using *ggplot2* (Wickham, 2016), (*ggpubr*), (*sjPlot*), (*sjmisc*) and (*qqplotr*). All the sperm motility data were analysed using linear mixed effect models (LMMs) and generalised linear mixed effect models (GLMMs) in *lme4* (Bates et al., 2015). To determine the error distributions, the relationship between the variance and the mean of the response variable and the assumptions for data distribution were checked (Crawley, 2012). Models were fitted using restricted maximum likelihood (REML) methods to enable refinement and validation. Residuals from linear models were checked for violations of the assumptions of normality and homoscedasticity. Significance of fixed effects in LMMs were obtained using *t*‐tests with Satterthwaite's approximation for degrees of freedom implemented in *lmerTest* (Kuznetsova et al., 2017). Main effects, contrast analyses and interactions, when present, were extracted through the *emmeans* and *emtrends* functions. For all the variables analysed, a selection of the best model structure to explain each variable was conducted by comparing residual dispersion, model predictions, AICs and BICs for each of the computed models through the ‘summary’ function output and through *DHARMa* residual diagnostic. Additionally, performance between the different models was compared by using the ‘*anova*’ function. All datasets associated to this study are archived and available via dryad (Graziano et al., 2025).

#### 2.7.1 Sperm swimming behaviour in non‐sibling and sibling ovarian fluid

Correlation matrices for sperm performance traits generated by the CASA output were generated to investigate collinearity within sperm kinematic parameters. Traits from 5 to 60 s post activation were combined using principal component analysis (PCA) to derive linear combinations (principal components (PCs)) retaining as much information as possible about the original variables and explaining a high proportion of the total variance within the dataset. This step allowed us to control the collinearity and extrapolate broader sperm kinematics patterns and resulting components used as fixed factors in our analyses (see Figure S2 in Supporting Information). Following this, we applied a two‐step clustering procedure using the principal components (PCs) derived from the PCA to identify sperm subpopulations based on kinematic characteristics, following the method described by Martínez et al. (2006). Individual GLMMs and LMMs on the effect of relatedness on individual sperm parameters were still run, and model details and results are reported in the Supporting Information (Table S1). Sperm motility parameters including percent motile sperm MOT%, VSL, LIN, BCF and PROG were analysed using LMMs, whereas VAP, VCL and WOB were analysed using GLMMs (*glmmTMB*, Brooks et al., 2017) due to better residual diagnostics of these over LMMs. All the final mixed effect models performed included the relatedness between mates (non‐siblings or full siblings), the seconds post sperm activation (spa, 5 to 60s) and their interaction as fixed factors, and female and male ID as random factors (ID 1 to 7) with the three experimental sperm samples tracked per male nested by male ID. Random slopes were included for these experimental males to account for the fully factorial design.

#### 2.7.2 Fertilisation and hatching rates in sibling and non‐sibling crosses

The proportion of eggs successfully fertilised for each batch of eggs, as well as the final percentage of fertilised embryos that succeeded to hatch, were modelled through GLMMs. The final models included the relatedness between mates (non‐siblings or full siblings) as a fixed factor, and female and male ID as random factors (ID 1 to 7) with the two experimental replicates per male nested by male IDs. Additional models were run with the averaged data from the fertilisation trials to include sperm traits PCs together with relatedness as fixed factors, avoiding pseudo‐replication of PC values across technical replicates (see Table S3).

#### 2.7.3 Morphometrics

The comparison of fork length and body weight between non‐sibling and sibling offspring was modelled at 6–24 months post‐hatching through LMMs, following a similar approach as previous models. The final models included the relatedness between parents (non‐siblings or full siblings) as a fixed factor, and the tank ID as a random factor (three tanks for sibling crosses and three tanks for non‐sibling ones).

#### 2.7.4 Sperm competition experiments and microsatellite analyses

Microsatellite genotyping data were used to determine the percentage of offspring sired by each male in the sperm competition experiments. Paternity rates and genotypic identities of all the individuals were transferred to R Studio and analysed for subsequent analyses. Paternity per cent (%) was converted to logarithmic form before being analysed using GLMMs (*glmmTMB*, Brooks et al., 2017). The model included the relatedness between mates (acting either as non‐siblings or full siblings when paired to a specific female), the number of shared alleles at the MHC1 and MHC2 loci (0, 1 or 2) and all their possible interactions as fixed factors, and female and male ID as random factors (ID 1 to 7). Random slopes were included for these experimental males to account for the fully factorial design.

### 2.8 Ethics

Approval of the experimental protocol by the Norwegian Animal Research Authority was not required as the rearing conditions were as in standard Atlantic salmon farming. All welfare and use of experimental animals were performed in strict accordance with the Norwegian Animal Welfare Act. Nevertheless, all personnel involved in this experiment had undergone training approved by the Norwegian Food Safety Authority, which is mandatory for all personnel running experiments involving animals included in the Animal Welfare Act. In addition, all experimental protocols received approval from the Animal Welfare and Ethical Review Board at the University of East Anglia, in accordance with UK and European legislation.

## 3 RESULTS

**FIGURE 3:**
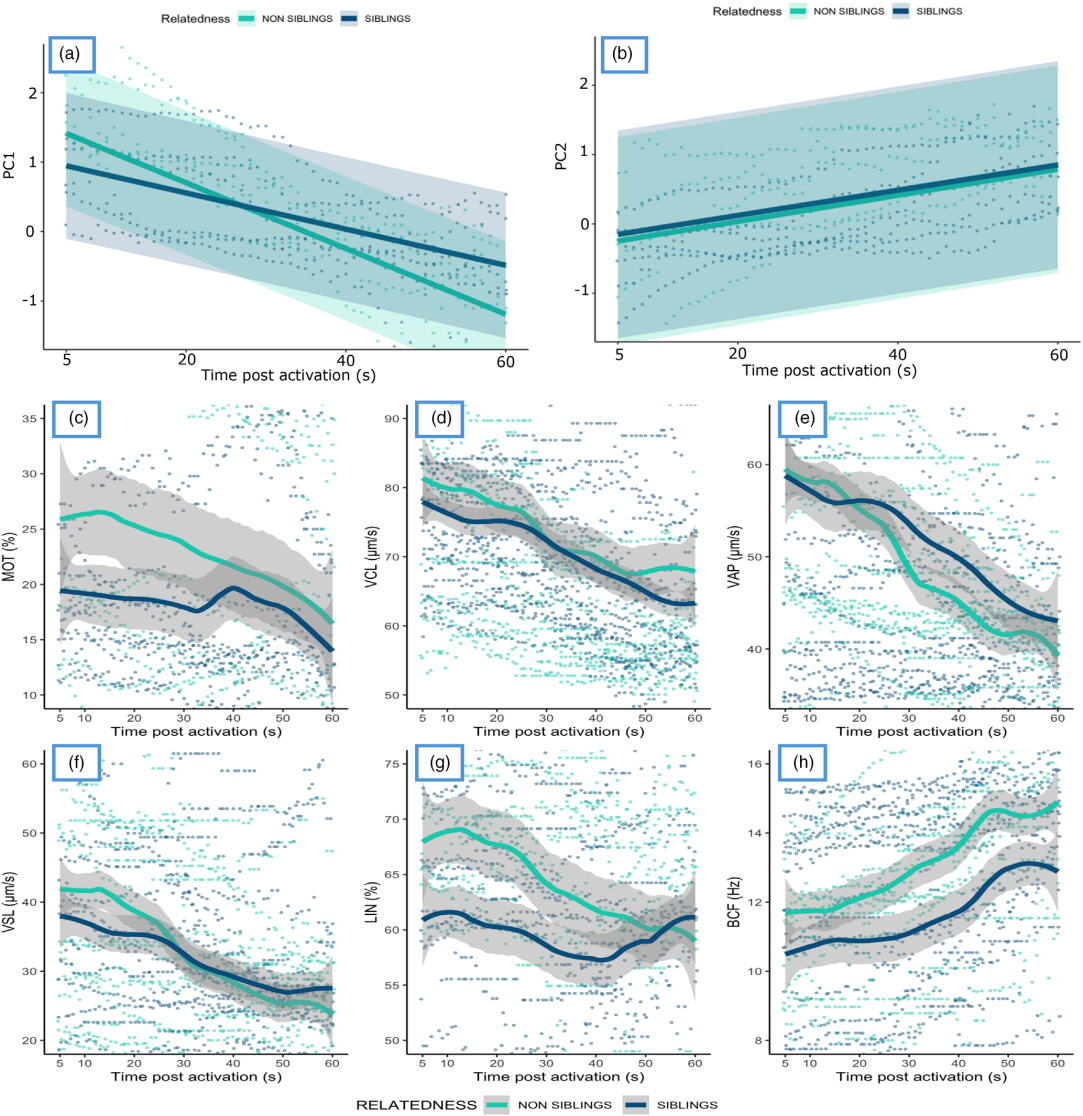
Sperm performance metrics in sibling (blue) and non‐sibling (green) ovarian fluid across time post‐activation (5–60 s) from 14 split‐design crosses (seven males × seven females). Panels (a and b) show mixed‐effects model fits with 95% confidence intervals (shaded areas) and raw individual data points for principal components PC1 and PC2, respectively. Panels (c and h) display loess‐smoothed average trends with shaded areas representing standard deviations (SD) and overlaid raw individual data points. Metrics include (c) sperm motility (MOT, %), (d) curvilinear velocity (VCL, μm/s), (e) average path velocity (VAP, μm/s), (f) straight line velocity (VSL, μm/s), (g) linearity (LIN, %) and (h) beat cross frequency (BCF, Hz).

The PCA on sperm traits returned two components (PCs) with eigenvalues greater than 1 that, when summed, explained 80.3% of the total variance of the dataset. These two PCs were used for analysis. Sperm PC1 was determined positively by VAP, VSL, WOB, PROG and BCF, while sperm PC2 was determined negatively by sperm VCL and MOT% (scree plots, details on loadings of the two PCs and variance explained are available in the Supporting Information Figure S1). Exposure of sperm to ovarian fluid from sibling females resulted in reduced motility over time (MOT%), lower velocity‐related parameters (VCL, VSL and VAP), lower linearity (LIN%), beat‐cross frequencies (BCF) and reduced wobble (WOB) compared to sperm exposed to ovarian fluid from non‐sibling females. Sperm exposed to sibling's ovarian fluid also achieved lower distances during their progression. This pattern was consistent among males and true for all the CASA parameters analysed and relevant for the prediction of fertilisation success (Gage et al., 2004; Gasparini et al., 2010; Wilson‐Leedy & Ingermann, 2007), with the biggest effects of relatedness observed for LIN and VSL (Figure 3, Figure S2, Table S1). Importantly, PC1 was negatively correlated with relatedness and with time post‐activation, and there was a significant interaction between the two (Figure 3, Figures S1 and S2, Table 1). Two‐step cluster procedures revealed the presence of three sperm subpopulations characterised respectively by positive values of PC1 and negative values of PC2 (Cluster 1); negative values for PC1 and positive values for PC2 (Cluster 2); negative values of PC1 and PC2 (Cluster 3). The three identified sperm subpopulations were distributed differently between sibling and non‐sibling crosses. Notably, Cluster 3, which was characterised by higher MOT% and VCL, was exclusively observed when sperm were swimming in non‐sibling ovarian fluid (*χ* ^2^ = 211.1, *p* < 2.2 × 10^−16^; see Figures S1 and S2, Table S2 for details).

**TABLE 1:** Mixed effect models (lmer in R) for sperm PC1 and PC2 in sibling or non‐sibling ovarian fluid from 5 to 60 s post activation.

PC1	
Random	Variance
Female ID	0.5280
Male ID	0.6408
Replicate: Male ID	0.8273

Fixed	Estimate	SE	CI	df1, df2	*t*‐value	*p*
Intercept	1.641e+00	4.985e‐01	−0.943, 0.036	1, 1.115e+01	3.205	**0.006**
Relatedness	−5.716e‐01	2.141e‐01	−0.943, 0.036	1, 19.367e+01	−2.670	**0.008**
Time post‐activation (s)	−4.736e‐02	3.505e‐03	−0.054, −0.039	1, 2.096e+03	−13.514	**<0.001**
Relatedness × Time post‐activation (s)	2.129e‐02	4.774e‐03	0.009, 0.030	1, 2.097e+03	4.460	**<0.001**

PC2	
Random	Variance
Female ID	1.415
Male ID	1.613
Replicate: Male ID	0.084

Fixed	Estimate	SE	CI	df1, df2	*t*‐value	*p*
Intercept	−3.408e‐01	6.976e‐01	0.545, 2.747	1, 1.151e+01	−0.489	0.634
Relatedness	9.883e‐02	1.940e‐01	−0.943, −0.036	1, 1.476e+01	0.510	0.618
Time post‐activation (s)	1.882e‐02	1.344e‐03	−0.054, −0.039	2.095e+03	14.001	**<0.001**
Relatedness × Time post‐activation (s)	−6.080e‐04	1.831e‐03	0.009, 0.030	1, 2.095e+03	−0.332	0.740

*Note*: The results are shown for a total of 14 split‐design crosses with seven males and seven females crossed pairwise. Estimates are provided with standard error (SE), confidence intervals (CI) and degrees of freedom (df1 = *k* − 1 and df2 = *n*_tot_ – *k*, where *k* is the number of treatment levels and *n*_tot_ is the total number of observations). Bold values indicate significance threshold: *p* < 0.05

**FIGURE 4:**
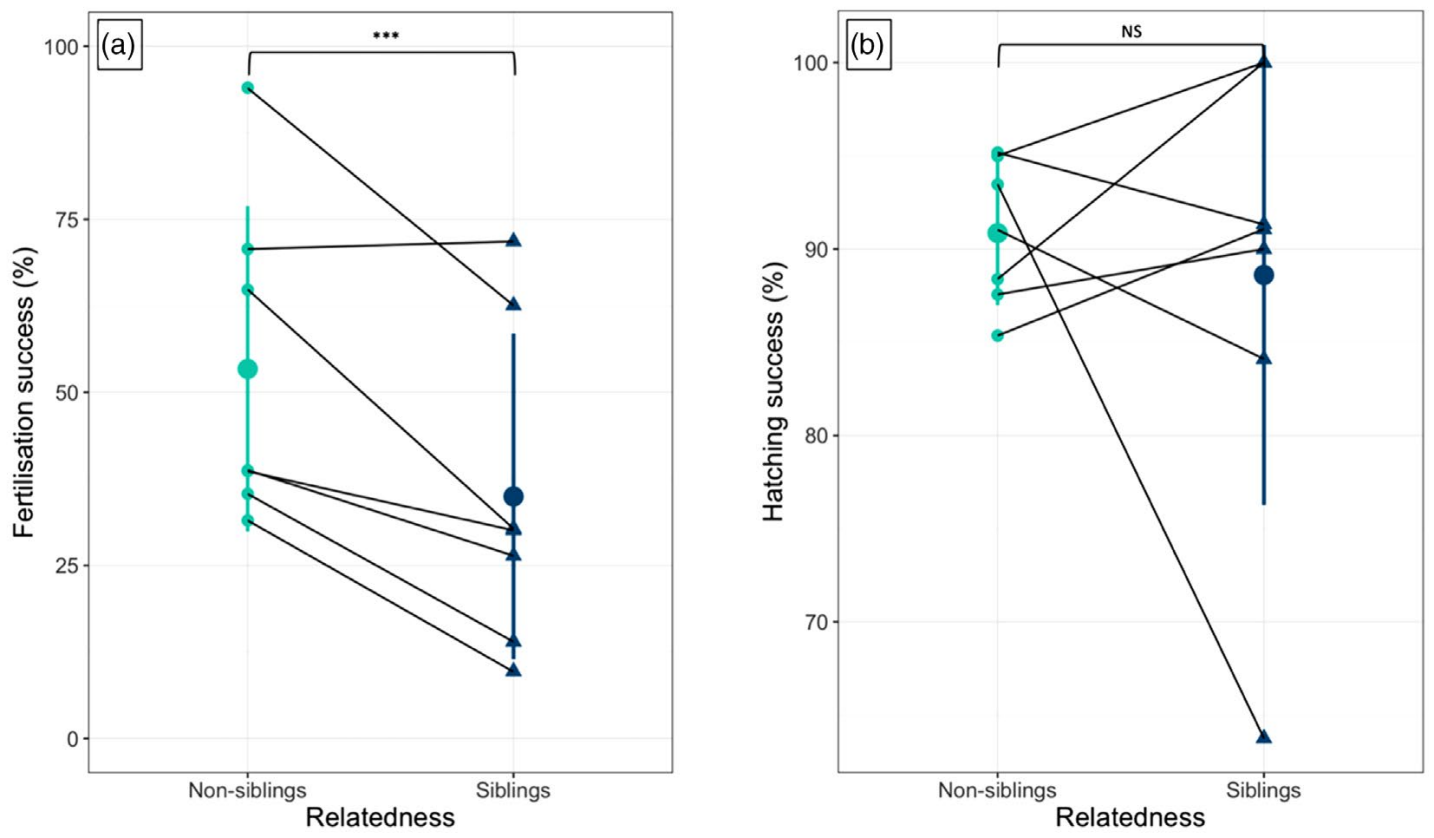
(a) Fertilisation and (b) hatching success (%) in non‐sibling (*N* = 7) and sibling crosses (*N* = 7). Data are displayed as means (big dots) ± standard deviation (SD, vertical bars); dark lines connect each experimental female used in the experiment (dots for non‐siblings and triangles for siblings). Significance threshold: ***p < 0.001.

**FIGURE 5:**
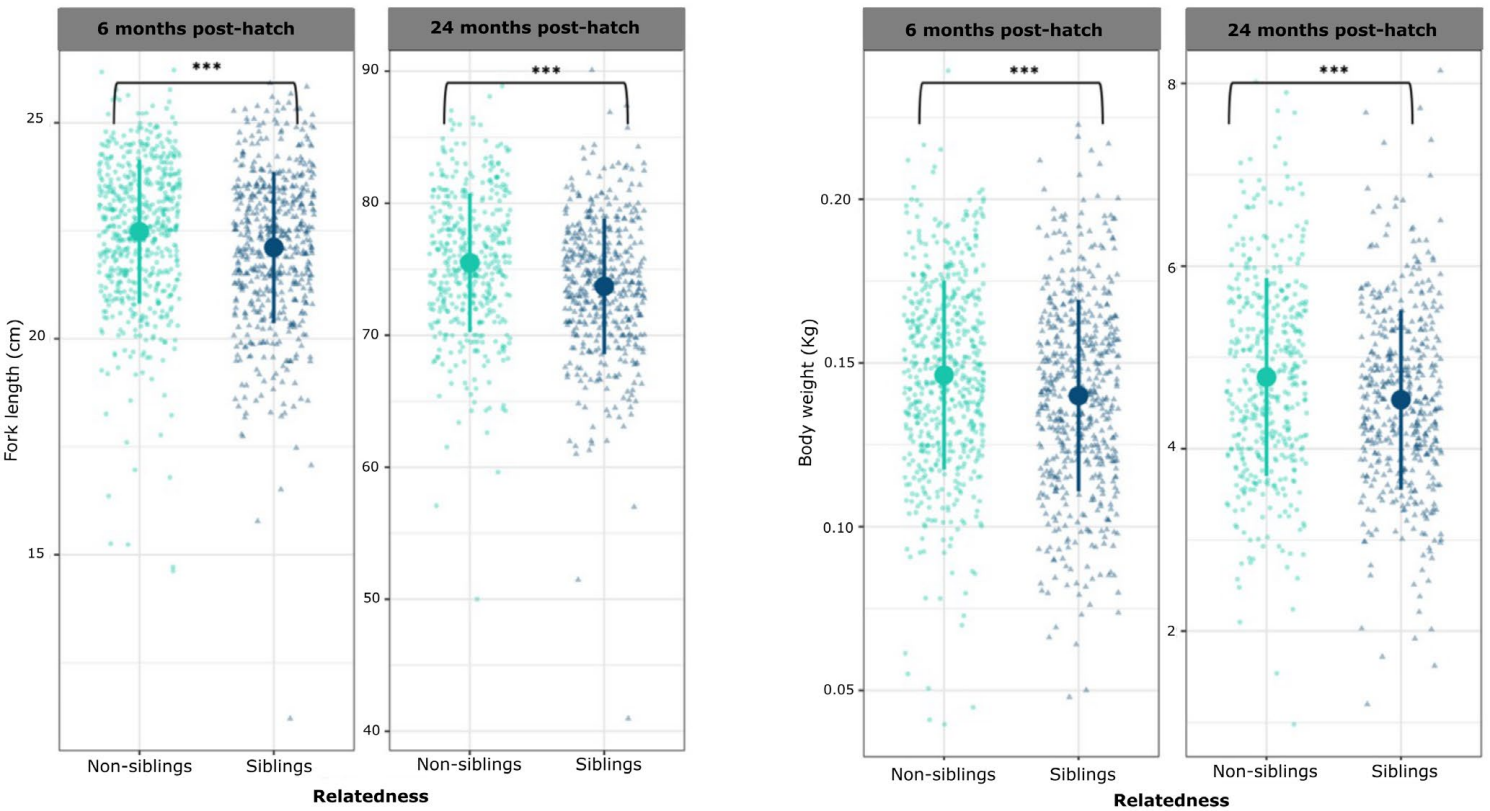
Comparison of fork length (cm) and body weight (kg) between sibling and non‐sibling offspring measured at 6 and 24 months post hatching. Sample sizes are 431 and 436 for sibling offspring and 576 and 544 for non‐sibling at 6 and 24 months respectively. The graphs display raw data (dots and triangles) and means (big dots) ± standard deviation (SD, vertical bars). Significance threshold: ***p < 0.001.

In line with the reduced sperm velocity parameters observed, fertilisation rates were lower in sibling crosses compared to non‐sibling crosses (35 ± 23.2% vs. 53 ± 26%, respectively, means ± SD; Figure 4a, Table 2). In contrast, once these eggs were fertilised, they led to an equal number of hatchlings in the two experimental groups (Figure 4b, Table 2), and the same was true for alevin or juvenile fish mortality over the duration of the experiment. When, in the mixed models, we accounted for sperm traits PCs to test the effects of relatedness on fertilisation and hatching success, we found a positive correlation between both PC1 and PC2 with hatching success, but not with fertilisation success that was again influenced only by siblingship (Table S2). Finally, alevins from sibling crosses showed lower body weight and length both at 6 and 24 months post hatching, with these differences being not dramatic but consistent among the 1120 fish analysed (Figure 5, Table 3). Interestingly, we found no effect of relatedness between males and females on paternity ratios in the sperm competition experiments. Similarly, in the competitive fertilisation trials, neither the number of shared alleles between males and females at the MHC1 and MHC2 loci nor the sperm principal components (PCs) were associated with siring success (see Figure S4 and Table S4 in the Supporting Information).

**TABLE 2:** Generalised liner mixed model (glmer in R) for fertilisation success (%) and for hatching success (%) in sibling or non‐sibling crosses.

Fertilisation success (%)	
Random	Variance
Female ID	39.06
Replicate: Male ID	54.11

Fixed	Estimate	SE	CI	df1, df2	*t*‐value	*p*
Intercept	53.39	8.22	35.20, 71.58	1, 8.70	−6.495	**<0.001**
Relatedness	−18.42	3.99	−27.10, −9.77	1, 9.79	−4.610	**<0.001**

Hatching success (%)	
Random	Variance
Female ID	12.33
Male ID	2.39

Fixed	Estimate	SE	CI	df1, df2	*t*‐value	*p*
Intercept	90.85	3.13	83.92, 97.78	1, 8.9	28.97	**<0.001**
Relatedness	−2.25	2.70	−8.05, 3.56	1, 13.05	−0.81	0.43

*Note*: The results are shown for a total of 14 split‐design crosses with seven males and seven females crossed pairwise. Estimates are provided with standard error (SE), confidence intervals (CI) and degrees of freedom (df1 = *k* − 1 and df2 = *n*_tot_ – *k*, where *k* is the number of treatment levels and *n*_tot_ is the total number of observations). Bold values indicate significance threshold: *p* < 0.05

**TABLE 3:** Linear mixed effect model (lmer in R) for changes in body weight (kg) and length (cm) in sibling or non‐sibling (Relatedness) crosses at sampled at 6 and 24 months post‐hatch.

Body weight (kg) 6 months post hatch	
Random	Variance
Tank	<0.001

Fixed	Estimate	SE	CI	df1, df2	*t*‐value	*p*
Intercept	0.14	0.001	0.143, 0.149	1, 1116	120.87	**<0.001**
Relatedness	−0.006	0.001	−0,010, 0.002	1, 1116	−3.63	**<0.001**

Fork length (cm) 6 months post hatch	
Random	Variance
Tank	**<0.001**

Fixed	Estimate	SE	CI	df1, df2	*t*‐value	*p*
Intercept	−22.48	0.07	22.34, 22.66	1, 1116	316	**<0.001**
Relatedness	−0.36	0.01	−0.56, −0.16	1, 1116	−3.59	**<0.001**

Body weight (kg) 2 years post hatch	
Random	Variance
Tank	0.0168

Fixed	Estimate	SE	CI	df1, df2	*t*‐value	*p*
Intercept	4.79	0.09	4.61, 4.96	1, 878	53.34	**<0.001**
Relatedness	−0.26	0.07	−0,40, −0.13	1, 878	−3.78	**<0.001**

Fork length (cm) 2 years post hatch	
Random	Variance
Tank	0.45

Fixed	Estimate	SE	CI	df1, df2	*t*‐value	*p*
Intercept	75.52	0.46	74.62, 76.43	1, 878	163.65	**<0.001**
Relatedness	−1.85	0.34	−2.53, −1.17	1, 878	−5.33	**<0.001**

*Note*: Models include the random factor for replicate experimental tank per relatedness status. Estimates are provided with standard error (SE), confidence intervals (CI) and degrees of freedom (df1 = *k* − 1 and df2 = *n*_tot_ – *k*, where *k* is the number of treatment levels and *n*_tot_ is the total number of observations). Bold values indicate signficance threshold: *p* < 0.05

## 4 DISCUSSION

We observed a clear effect of ovarian fluid from sibling and non‐sibling females on sperm performance and fertilisation success in non‐competitive trials. Sperm PC1, motility, velocity and linearity, as well as fertilisation success, were all higher in the ovarian fluid of non‐sibling females. In addition, fish resulting from sibling crosses were smaller than those from the non‐sibling crosses throughout the experiment. These findings suggest that, while inbreeding depression is possible, as indicated by the reduced size of juvenile and adult fish six and 24 months after hatching, inbreeding avoidance mechanisms have evolved in Atlantic salmon at the gamete level, mediated by the interaction between sperm and ovarian fluid. However, when sperm from sibling and non‐sibling males were competing simultaneously for the same egg batch, we found no influence of this on paternity. This opens the possibility that, in the presence of intense competition on the spawning grounds, factors other than relatedness may play a major role in determining the reproductive outcome. Overall, our results suggest that, while the ovarian fluid may provide a pre‐zygotic barrier to inbreeding, this barrier is relatively weak and may be overcome in situations of sperm competition.

### 4.1 Post‐mating pre‐zygotic mate choice

Our current knowledge of the mechanisms underpinning kin‐recognition at the gamete level comes mainly from internally fertilising species and mammals in particular (de Boer et al., 2021). Most of these studies tested inbreeding avoidance across varying levels of genetic similarity (e.g. unrelated, half‐sibling crosses and inbred and outbred individuals; (Pike et al., 2021)). In house mice (*Mus musculus*), a fertilisation bias against genetically related males suggests egg‐driven sperm selection towards unrelated males (Ee et al., 2015). Similarly, in houbara bustards (*Chlamydotis undulata*), for example, unrelated males sired more offspring than cousins or half‐siblings, but this effect was explained through differential embryo mortality among the crosses rather than by the presence of a pre‐zygotic barrier to inbreeding (Vuarin et al., 2018). This represents a post‐zygotic inbreeding avoidance mechanism, whereby selection occurs after fertilisation through reduced viability or increased abortion of inbred embryos. Here, we use the term ‘post‐zygotic’ specifically to refer to such processes acting after zygote formation, rather than to post‐copulatory but pre‐zygotic phenomena such as sperm selection or ovarian fluid effects. In the internally fertilising Trinidadian guppy (*Poecilia reticulata*), the ovarian fluid of unrelated females enhanced sperm swimming performance compared to the ovarian fluid of related females (Gasparini & Pilastro, 2011). In this study, paternity in competition trials was biased towards unrelated males, which contrasts with our results. One possible explanation for these contrasting results is the fundamentally different reproductive tactics and life histories of the two species: In externally fertilising species, females are expected to favour stronger pre‐zygotic mechanisms, whereas live‐bearers should theoretically allocate more in post‐zygotic mechanisms (Zeh & Zeh, 2000, 2001, 2006). In our study, we observed that sperm in non‐sibling fluid showed faster and straighter swimming trajectories during the first seconds post sperm activation. This occurred at the expense of longevity (Kholodnyy et al., 2020), and directionality at the end of the tracking at 60 s. The effect, however, was detected only in non‐competitive trials. These altered sperm velocities and trajectories could have translated in lower reproductive success in the sibling crosses, further confirming that sperm velocity is a prime determinant of fertilisation success in salmon (Gage et al., 2004) as well as in other fish species like the green swordtail *Xiphophorus helleri* (Gasparini et al., 2010) and the domestic pig *Sus domesticus* (Fernández‐López et al., 2022).

The absence of a fertilisation bias in our competitive trials contrasts with the findings of Gasparini and Pilastro (2011) and may reflect fundamental differences in fertilisation mode and gamete interaction context. In internal fertilisers, post‐copulatory mechanisms such as cryptic female choice may exert more precise control over sperm use, while in externally fertilising species, fertilisation success is likely shaped more by physical and chemical interactions in the immediate gamete environment. Moreover, sperm competition may obscure or override subtle compatibility effects observed under non‐competitive conditions. These differences underscore the need for further studies comparing competitive and non‐competitive fertilisation across reproductive modes. How the ovarian fluid differentially influences sperm by kinship remains unknown. This could involve signalling peptides dispersed within the fluid and receptors on the sperm, as found in mammals (Carlstedt et al., 1983; Paradisi et al., 2000; Spehr et al., 2003). Although MHC peptides have been proposed as feasible candidates in such mechanisms in other external fertilisers, their identification in the ovarian fluid has still to be confirmed and so does the MHC expression on sperm. However, our results suggest that if these components are present in the ovarian fluid, they are unlikely to be involved in this mechanism; at least for the domesticated strain of salmon used here. Other reproductive proteins playing a role in the fertilisation process could be more important (Swanson & Vacquier, 2002). Future research should identify peptides influencing sperm behaviour, such as the decapeptide speract in sea urchins (Wood et al., 2007). In zebrafish (*Danio rerio*), ovarian fluid affects sperm differently across males, suggesting cryptic female choice (Poli et al., 2019), though mechanisms are unclear. Understanding the mechanistic series of events leading to a differential behaviour of sibling or non‐sibling sperm in the presence of ovarian fluid could therefore be a priority for future research. It would also be interesting to investigate whether the dynamics of mate selection in domesticated salmon are also applicable to their wild conspecifics, and what the consequences of this could be following salmon escapes in the natural environment.

### 4.2 MHC‐based mate choice and gamete fusion

We tested how fertilisation, hatching rates and paternity under sperm competition were influenced by the parental relatedness. Moreover, we aimed to determine whether the number of alleles shared between partners at the MHC1 and MHC2 loci had an effect on the siring potential of males. These two markers have been found to be involved in mate choice in a variety of species (Jordan & Bruford, 1998; (Chinook salmon, *Oncorhynchus tshawytscha*) Neff et al., 2008; (house mouse, *Mus musculus*) Penn & Potts, 1998; (*O. tshawytscha*) Pitcher & Neff, 2006; (*M. musculus*) Potts et al., 1991; (stickleback, *G. aculeatus*) Reusch et al., 2001; (Arctic charr, *Salvelinus alpinus*) Skarstein et al., 2005; (Humans) Wedekind et al., 1995, (whitefish, *Coregonus lavaretus*) Wedekind et al., 1996). However, their mechanistic role in mate choice and kin recognition is still highly debated (Ziegler et al., 2005), and we therefore tried to test the idea in the context of inbreeding avoidance mechanisms between sibling and non‐sibling crosses in an externally fertilising animal model. Our experimental design using all males multiple times in both sibling and non‐sibling roles allows us to reject an involvement of any microsatellite loci used in this study in post‐mating mate choice. We discuss our findings in view of previous experimental results.

Pre‐zygotic mechanisms of kin‐recognition in external fertilisers are still relatively poorly understood. In Atlantic salmon, for example, sperm competition experiments between males and females with similar or dissimilar MHC1 alleles provided evidence of a fertilisation bias towards males with dissimilar MHC alleles (Yeates et al., 2009). In contrast, experimental testing for MHC*2*‐based gamete fusion at the haploid level in Atlantic salmon showed no evidence for non‐random fusion (Promerová et al., 2017). In the brown trout (*S. trutta*), females favoured mates with an intermediate level of MHC1 similarity, but the exact mechanisms of mate choice and the relative importance of pre‐ and post‐mating pre‐zygotic mechanisms were not determined (Forsberg et al., 2007). Our contrasting results with the previous study on MHC1 (Yeates et al., 2009) could be explained by the fact that we paired our mates according to their sibling non‐sibling status, rather than according to their MHC variability. This is similar to what Promerová et al. (2017) did and may explain why neither found any evidence for a role of MHC dissimilarity. Such a mechanism may only materialise in comparisons between mating partners at opposite ends of the MHC similarity spectrum and, in fact, the interaction between relatedness and MHC may make the detection of a non‐random mating pattern hard. Another possible explanation for the discrepancy in the results could be the use of wild (Yeates et al., 2009) versus domesticated (this study) populations. After decades of artificial selection due to aquaculture management (Campton, 2004; Glover, Solberg, et al., 2017), we cannot exclude that MHC‐based kin recognition mechanisms could have been inadvertently altered, as has been described for a wide range of other traits (Glover, Solberg, et al., 2017). Finally, in a whitefish (*Coregonus* sp.), there was also no evidence for MHC‐based sexual selection (Wedekind et al., 2004) and the authors argued that MHC‐based selection likely did not evolve in fish due to the higher costs of non‐random gamete fusion in externally fertilising species. In externally fertilising species, gametes are released into a hostile environment and quickly have to come into contact, therefore reducing the chances for assortative mating. In addition, selection for assortative mating could be weaker because of the large amounts of eggs and the lower allocation per egg compared to mammals, and because a generally lower risk of inbreeding might have not selected towards tactics to avoid its detrimental effects (Wedekind et al., 2004). Our results could be an interesting counterpoint to other studies suggesting that polyandry may provide females with opportunities to avoid inbreeding by mating with multiple males (Tregenza & Wedell, 1998, 2000). In view of our findings, it could be asked whether sperm competition may be costly to females, as it appears to weaken barriers against inbreeding.

By controlling the starting number of eggs per female and precisely determining the number of fertilised eggs within minutes of contact between male and female gametes, we are confident that the difference in reproductive outcomes observed in the first set of experiments (homospermic fertilisation trials) is not caused by differential early embryo mortality between non‐sibling and sibling crosses. One of the motivations in support of highly variable MHC complexes is linked to the enhanced capacity of ensuring resistance to a broader spectrum of fast‐evolving parasites, bacteria and viruses (Agbali et al., 2010; Wedekind et al., 2004). We found no differential hatching success, embryonic or egg to adult survival in sibling or non‐sibling crosses in either experiment, suggesting no MHC‐mediated improved survival in our experiment. However, this is a cautious conclusion, as we did not specifically test for this, and because our eggs and embryos developed in controlled aquaculture settings.

### 4.3 Inbreeding depression and inbreeding avoidance mechanisms

Intriguingly, the presence of a pre‐zygotic preference for unrelated sperm within the ovarian fluid, in combination with a small but statistically significant level of inbreeding depression observed in inbred offspring, would suggest that the barrier posed by the ovarian fluid is overall weak. This contrasts with the widely assumed concept that inbreeding depression should drive the evolution of inbreeding avoidance mechanisms, but new views, referred to as the ‘inbreeding paradox’ (Reid et al., 2015), justify the coexistence of inbreeding depression without or in the presence of a weak avoidance mechanism (Kokko & Ots, 2006; Szulkin et al., 2013). This is not a unique occurrence in nature, and comparable results have been found in different species, including the house sparrow (*Passer domesticus*) and the song sparrow (*Melospiza melodia*) (Billing et al., 2012; Olson et al., 2012; Reid et al., 2015). Ebel and Phillips (2016) highlighted that intrinsic differences between males and females lead to sex‐specific consequences of inbreeding, which may further complicate the predictions of sexual conflict over inbreeding. The concept of optimal inbreeding, as discussed by Puurtinen (2011), suggests that some degree of inbreeding may evolve under various conditions of inbreeding depression, even when mate choice is influenced by the sex with lower inbreeding tolerance. Males may tolerate inbreeding due to potential reproductive benefits, while females may prioritise genetic diversity to enhance offspring fitness, leading to conflicting selective pressures.

It is worth noting that, while we observed significant statistical differences in length and weight between sibling and non‐sibling offspring, effect sizes were rather small, which could point to their biological relevance, although slight variations in size could confer advantages in survival‐related aspects in salmonids. For instance, larger juvenile size has been correlated with higher survival rates, enhanced ability to compete for resources and increased resistance to predation (Metcalfe et al., 1995, 2003; Reid et al., 2019). These advantages are more evident in natural environments, where competition for feeding territories and predator avoidance are critical for survival. Small differences in length and weight, amplified by environmental pressures, can significantly influence long‐term success by conferring slight but crucial competitive edges (Thorpe, 1989). Moreover, in hatchery‐reared populations, minimising growth variability is crucial to reduce hierarchical competition, which could otherwise lead to dominances negatively impacting overall growth and welfare (McCarthy et al., 2003). On the other hand, salmon, like other teleosts, can show compensatory growth phenomena also at later stages of their life (Damsgird & Dill, 1998; Maclean & Metcalfe, 2001). Therefore, we believe that further, and long‐term studies should investigate the biological significance of the observed differences in weight and length.

Our results are in line with a study focusing on haploid selection and finding no haploid MHC2‐based assortative gamete fusion in Atlantic salmon (Promerová et al., 2017). Similarly, in brown bears (*Ursus arctos*), there is no evidence that females display mating preferences according to MHC similarity. The authors also found no association between the reproductive success of a male and the MHC alleles he carried, providing no support for any role of mate choice in shaping MHC polymorphism in this species (Kuduk et al., 2014) There is in fact evidence that mate preferences can operate not exclusively at the level of key individual loci and in a directional way, but that instead favourable allelic combinations could be assessed on a broader genomic scale (Mays & Hill, 2004; Neff & Pitcher, 2005; Tregenza & Wedell, 2000). For instance, 55 SNPs showing a signature of sexual selection and 611 SNPs involved in differential viability have been identified in the Gulf pipefish, *Syngnathus scovelli* (Flanagan et al., 2016). Similarly, another genome‐wide screening study in the plant *Mimulus guttatus* reported several hundreds of SNPs exhibiting a signature of viability selection (Monnahan et al., 2021), suggesting that although a few loci can show greater effects, mate choice operates on multiple levels. A genome‐wide approach and the inclusion of more genetically diverse (wild) salmon strains may be needed to provide more details about the potential for the evolution of inbreeding mechanisms in this species.

## 5 CONCLUSIONS

Our results show that the ovarian fluid from a domesticated salmon strain can differentially regulate sperm swimming performance according to relatedness, but that this barrier does not seem to be strong enough to bias the paternity outcome and reduce inbreeding depression when unrelated and related males are simultaneously competing to fertilise a set of eggs. These results are in contrast with some other studies using wild salmonids and suggest that a strong inbreeding avoidance mechanism and/or an MHC recognition system could not evolve or was lost in the domesticated salmon strain used here, despite the mild decrease in fitness observed after inbreeding. We also suggest that a combination of pre‐ and post‐zygotic mechanisms could mask the real strength of a bias in reproductive outcomes between mating partners of varying relatedness; and that further studies should look towards a genome‐wide enhancement of heterozygosity or locally adapted traits, rather than focus on specific alleles.

## AUTHOR CONTRIBUTIONS

Marco Graziano: data curation, formal analysis, investigation, methodology, validation, visualisation, writing—original draft, writing—review and editing; Monica F. Solberg: conceptualisation, funding acquisition, investigation, project administration, resources, writing—review and editing; Kevin A. Glover: funding acquisition, project administration, resources, supervision, writing—review and editing; David Murray: formal analysis, investigation, writing—review and editing; Martin Taylor: formal analysis, review and editing; Anne Grete Eide Sørvik: formal analysis and validation; Simone Immler: methodology, supervision, validation, writing—review and editing; Matthew J.G. Gage: conceptualisation, funding acquisition, methodology, project administration, resources, supervision, writing—original draft. All authors gave final approval for publication and agreed to be held accountable for the work performed therein.

## FUNDING INFORMATION

This research was supported by grants available to Matthew J.G. Gage from the UK Research and Innovation Biotechnology and Biological Sciences Research Council and by grants available to Kevin A. Glover and Monica F. Solberg and financed by the Norwegian Department for Industry and Fisheries (project INTERACT). The Open Access funding was provided by the University of East Anglia.

## CONFLICT OF INTEREST STATEMENT

We declare we have no competing interests.

## Supporting information


**Table S1.** Mixed effect models (lmer and glmmTMB in R) for sperm linearity (%), sperm beat cross frequency (Hz), sperm wobble (%) and sperm progression (μm) in sibling or non‐sibling ovarian fluid from 5 to 60s post activation.
**Table S2.** Descriptive statistics of the sperm subpopulations identified following the two‐steps clustering procedure and their distribution across non sibling and sibling groups.
**Table S3.** Generalised liner mixed model (glmmTMB in R) for (log) fertilisation success (%) and for hatching success (%) in sibling or non‐sibling crosses.
**Table S4.** Generalised mixed effect model (glmmTMB in R) for paternity (log) by non‐sibling (*n* = 7) and sibling fathers (*n* = 7) within each of the trios.
**Figure S1.** The left panel (A) displays the results of the Principal Component Analysis (PCA) conducted on sperm motility variables. Each arrow represents a variable's contribution to the principal components, with the direction and length indicating the variable's influence. PC1 accounts for 59.1% of the variance, primarily influenced by variables such as VSL, VAP, WOB, BCF and PROG while PC2 explains 22.2% of the variance, driven by MOT and VCL. The colour gradient of the arrows represents the contribution of each variable, with warmer colours (red and orange) indicating higher contributions. The right panels summarize the contribution of variables to PC1 (B) and PC2 (C) and the entity and sign of each of the loadings. The dashed red lines indicate the threshold for significance, highlighting variables that contribute substantially to each dimension.
**Figure S2.** (A) Mean contribution of PC1 and PC2 to each cluster following the two‐steps cluster analysis. (B) Cluster membership (%) for sperm activated in 100% non‐sibling and sibling ovarian fluid.
**Figure S4.** Percentage of hatched offspring sired by non‐sibling (green, *n* = 7) or sibling males (blue, *n* = 7) in paired sperm competition assays with females (*n* = 7). Data shown represent mean ± standard deviation (SD).
**Figure S5.** Input parameters for the CASA automated sperm analyses used in this study.

## Data Availability

Datasets and scripts associated with this study are publicly available on the DRYAD repository (Graziano et al., 2025) under the unique digital object identifier DOI: https://doi.org/10.5061/dryad.rbnzs7hqp.
