# Supplementary material for: Limited inbreeding avoidance at the gamete level despite inbreeding depression in Atlantic salmon (Salmo salar)
Source: J Anim Ecol. 2025 Sep 3;94(11):2220–39. doi: 10.1111/1365-2656.70123 (PMC12586779; doi:10.1111/1365-2656.70123)
Supplement: Supplementary file 1 — Table S1. Mixed effect models (lmer and glmmTMB in R) for sperm linearity (%), sperm beat cross frequency (Hz), sperm wobble (%) and sperm progression (μm) in sibling or non‐sibling ovarian fluid from 5 to 60s post activation. Table S2. Descriptive statistics of the sperm subpopulations identified following the two‐steps clustering procedure and their distribution across non sibling and sibling groups. Table S3. Generalised liner mixed model (glmmTMB in R) for (log) fertilisation success (%) and for hatching success (%) in sibling or non‐sibling crosses. Table S4. Generalised mixed effect model (glmmTMB in R) for paternity (log) by non‐sibling (n = 7) and sibling fathers (n = 7) within each of the trios. Figure S1. The left panel (A) displays the results of the Principal Component Analysis (PCA) conducted on sperm motility variables. Each arrow represents a variable's contribution to the principal components, with the direction and length indicating the variable's influence. PC1 accounts for 59.1% of the variance, primarily influenced by variables such as VSL, VAP, WOB, BCF and PROG while PC2 explains 22.2% of the variance, driven by MOT and VCL. The colour gradient of the arrows represents the contribution of each variable, with warmer colours (red and orange) indicating higher contributions. The right panels summarize the contribution of variables to PC1 (B) and PC2 (C) and the entity and sign of each of the loadings. The dashed red lines indicate the threshold for significance, highlighting variables that contribute substantially to each dimension. Figure S2. (A) Mean contribution of PC1 and PC2 to each cluster following the two‐steps cluster analysis. (B) Cluster membership (%) for sperm activated in 100% non‐sibling and sibling ovarian fluid. Figure S4. Percentage of hatched offspring sired by non‐sibling (green, n = 7) or sibling males (blue, n = 7) in paired sperm competition assays with females (n = 7). Data shown represent mean ± standard deviation (SD). Fi [file JANE-94-2220-s001.docx]

## Supplementary Material

*Table S1 Mixed effect models (lmer and glmmTMB in R) for sperm linearity (%), sperm beat cross frequency (Hz), sperm wobble (%) and sperm progression(μm) in sibling or non-sibling ovarian fluid from 5 to 60s post activation. The results are shown for a total of 14 split-design crosses with seven males and seven females crossed pairwise. Estimates are provided with standard error (SE), confidence intervals (CI) and degrees of freedom (df1= k -1 and df2= n_tot_ – k, where k is the number of treatment levels and n_tot_ is the total number of observations).*

*
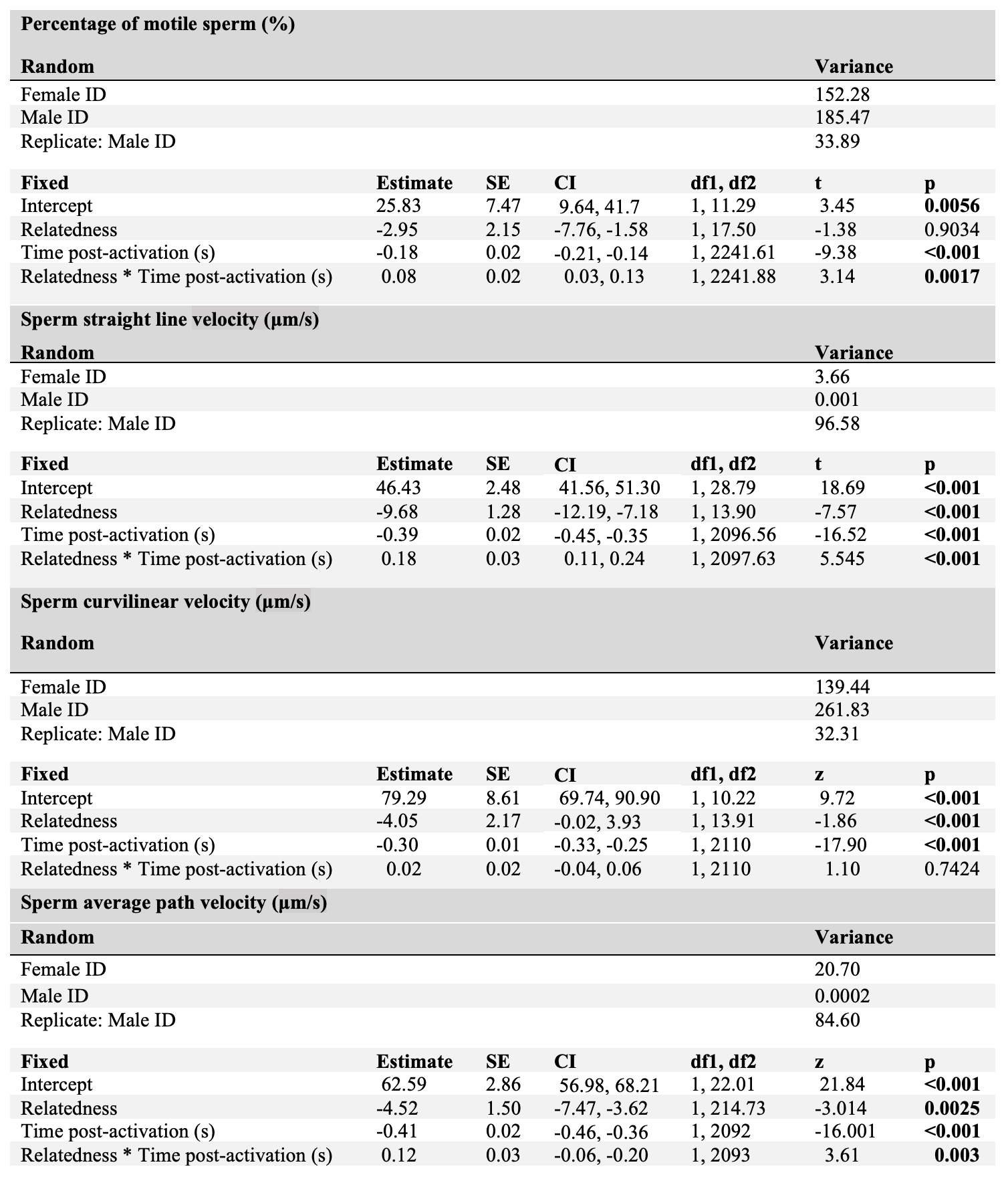
*


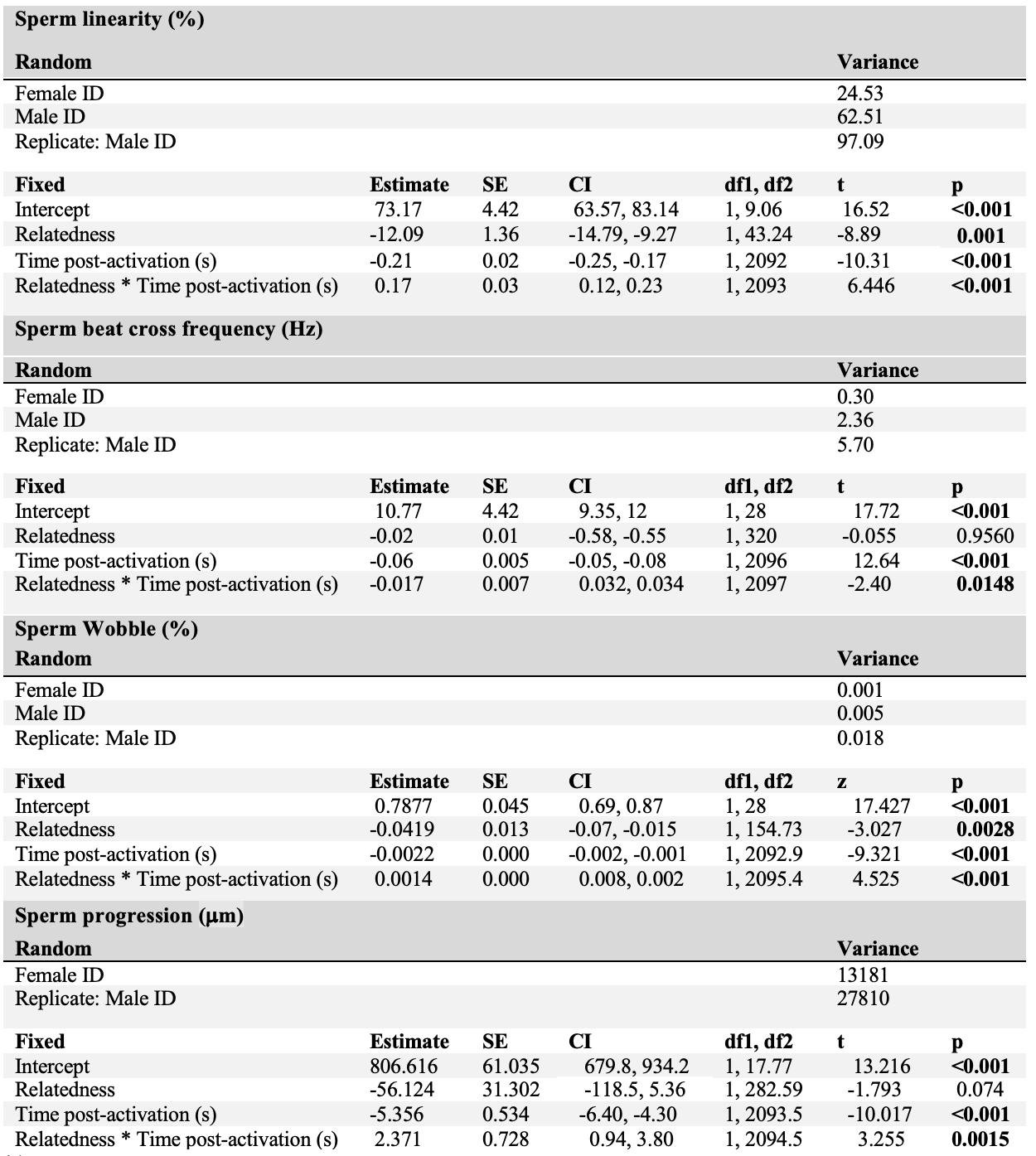


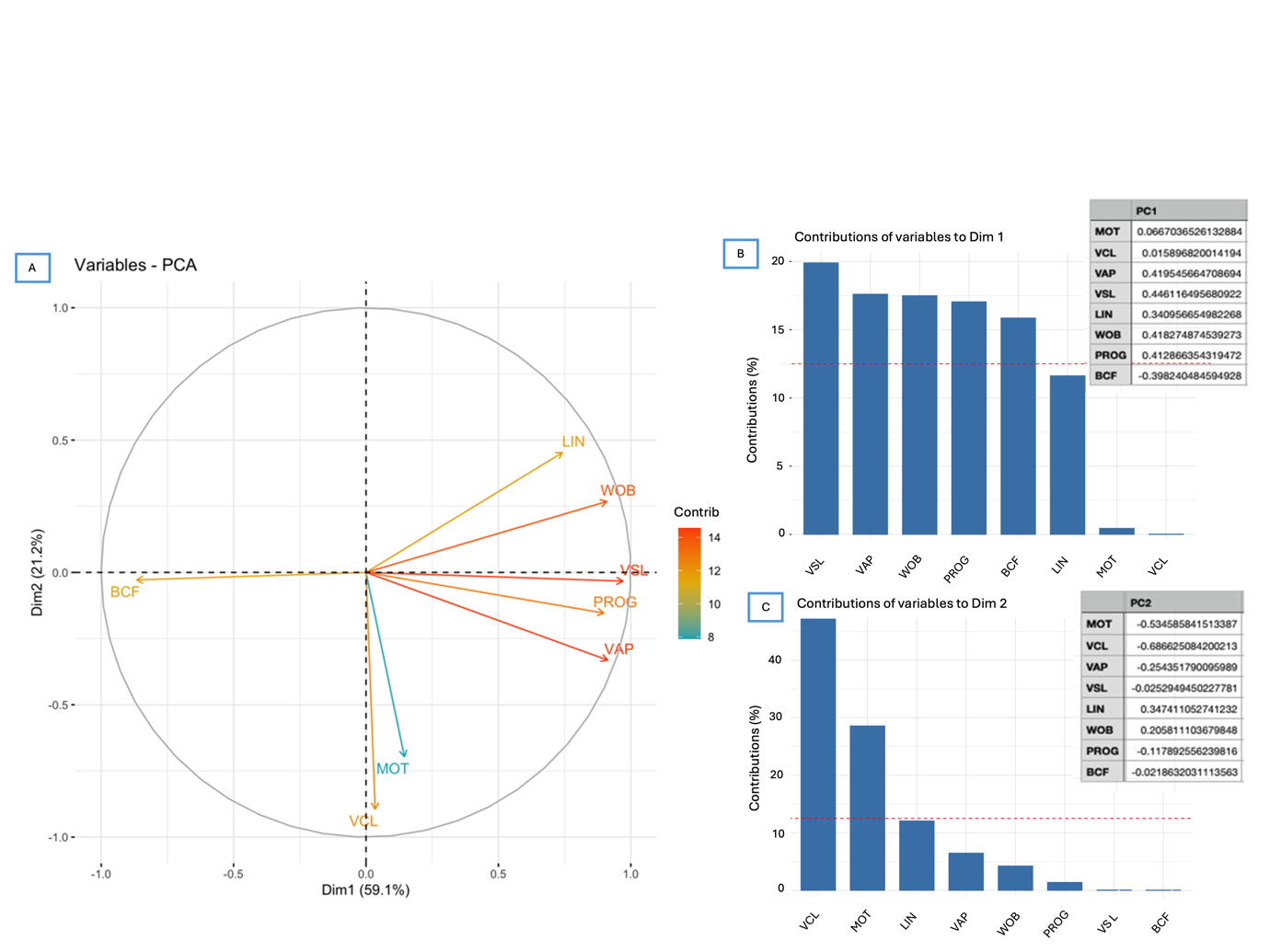


*Figure S1* *The left panel (A) displays the results of the Principal Component Analysis (PCA) conducted on sperm motility variables. Each arrow represents a variable's contribution to the principal components, with the direction and length indicating the variable's influence. PC1 accounts for 59.1% of the variance, primarily influenced by variables such as VSL, VAP, WOB, BCF and PROG while PC2 explains 22.2% of the variance, driven by MOT and VCL. The colour gradient of the arrows represents the contribution of each variable, with warmer colours (red and orange) indicating higher contributions. The right panels summarize the contribution of variables to PC1 (B) and PC2 (C) and the entity and sign of each of the loadings. The dashed red lines indicate the threshold for significance, highlighting variables that contribute substantially to each dimension.*


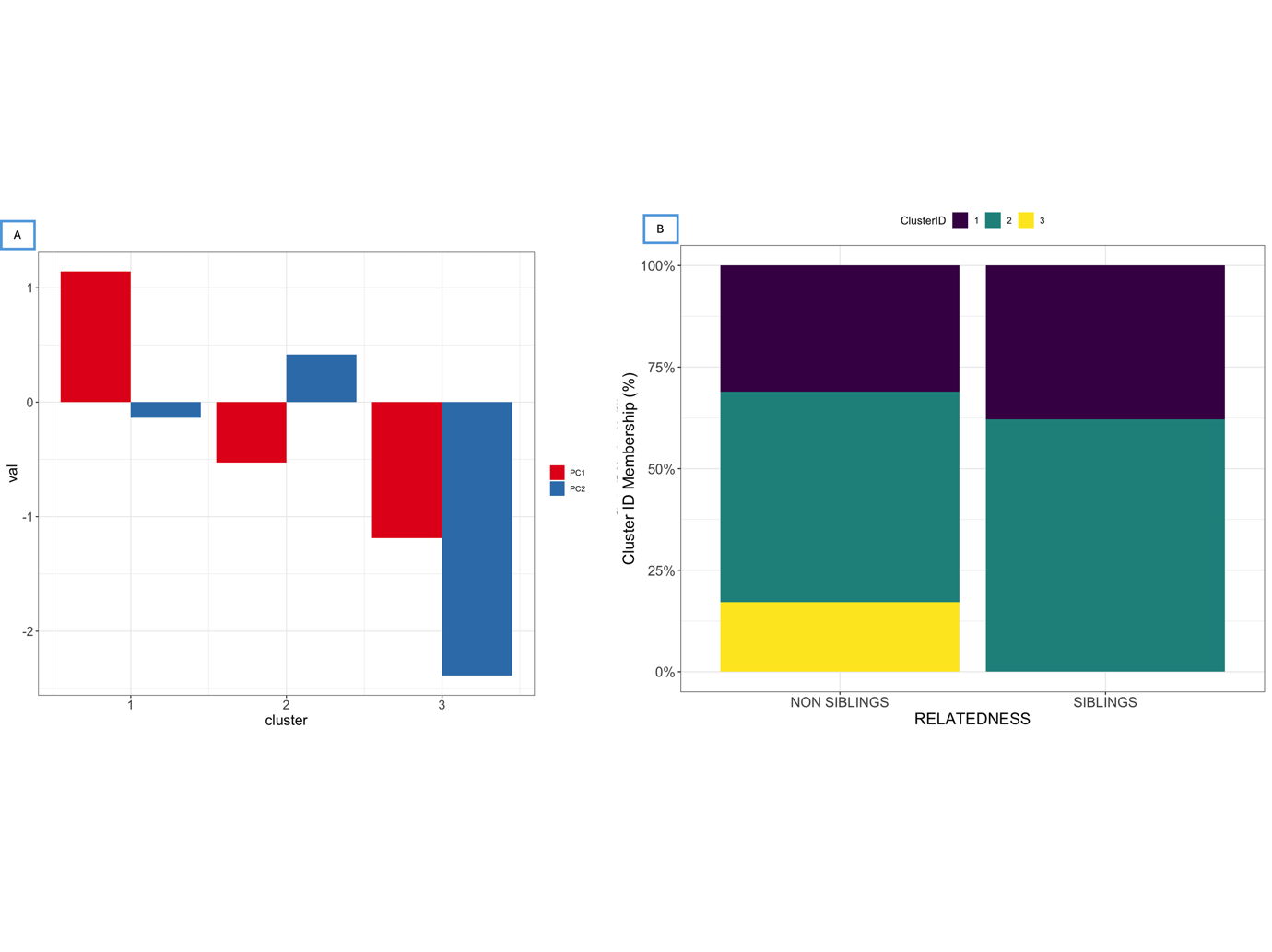


*Figure S2. (A)Mean contribution of PC1 and PC2 to each cluster following the two-steps cluster analysis. (B) Cluster membership (%) for sperm activated in 100% non-sibling and sibling ovarian fluid.*

*Table S2 Descriptive statistics of the sperm subpopulations identified following the two-steps clustering procedure and their distribution across non sibling and sibling groups.*


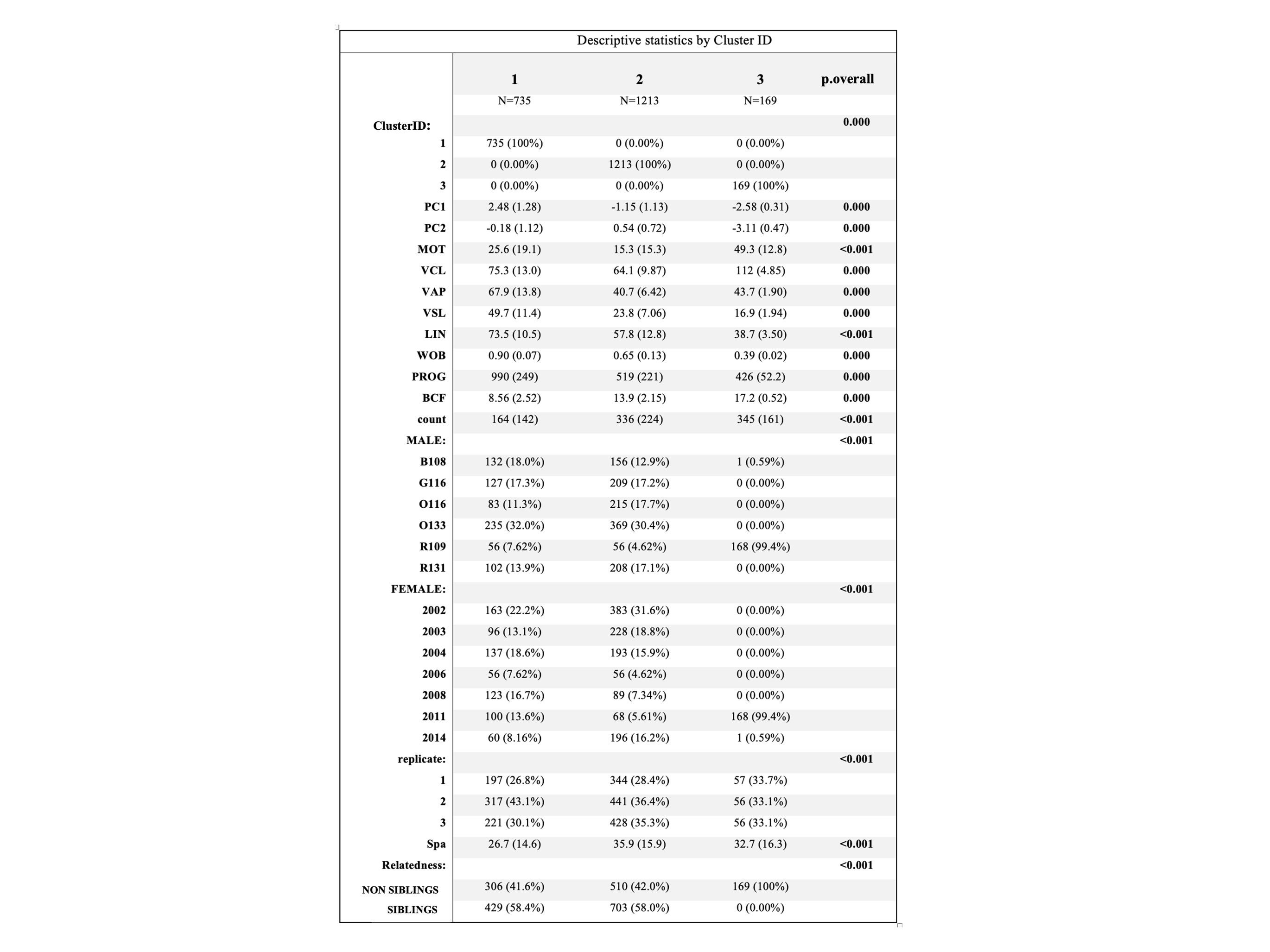


*Table S3 Generalised liner mixed model (glmmTMB in R) for (log) fertilisation success (%) and for hatching success (%) in sibling or non-sibling crosses. The results are shown for a total of 14 split-design crosses with seven males and seven females crossed pairwise. Estimates are provided with standard error (SE), confidence intervals (CI) and degrees of freedom (df1= k -1 and df2= n_tot_ – k, where k is the number of treatment levels and n_tot_ is the total number of observations).*

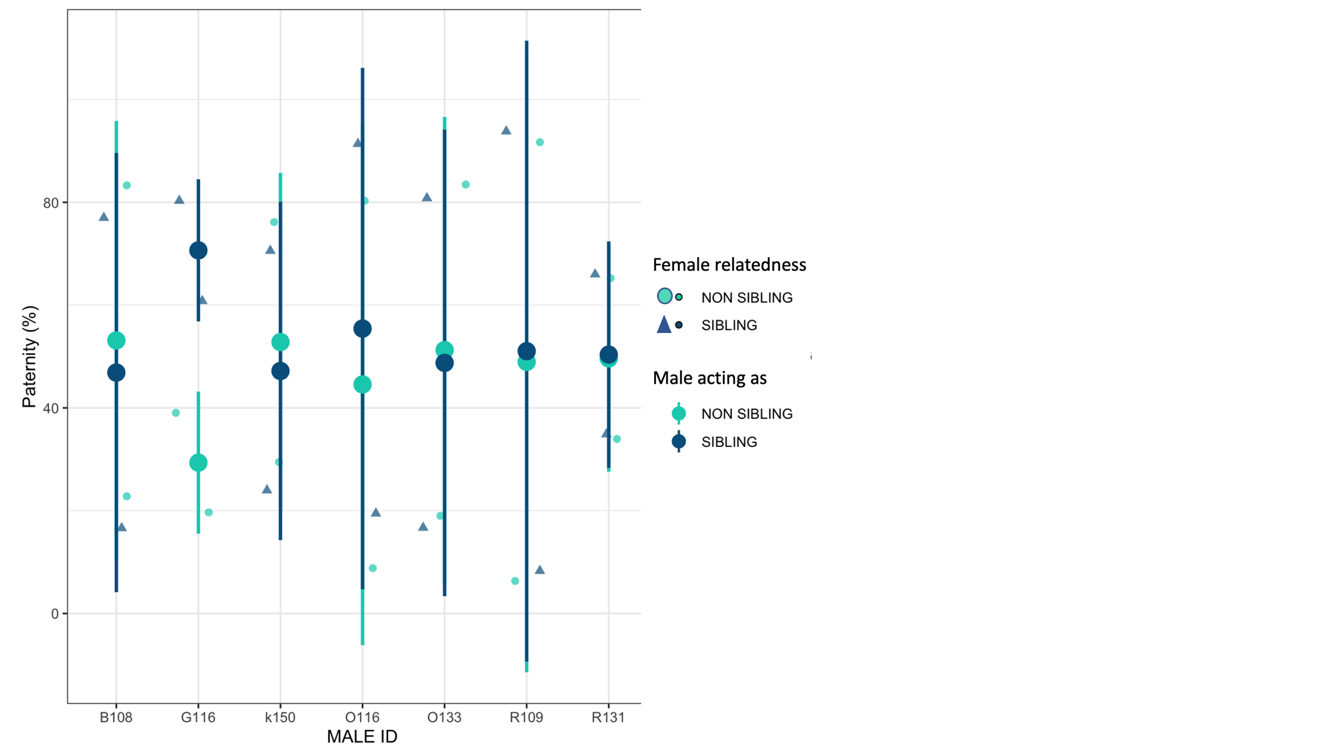


*Figure S4 Percentage of hatched offspring sired by non-sibling (green, n= 7) or sibling males (blue, n= 7) in paired sperm competition assays with females (n= 7). Data shown represent mean ± standard deviation (SD). Larger dots represent the averages across single experimental replicates which are instead represented as smaller dots for non-siblings and small triangles for sibling fish.*

*Table S4 Generalised mixed effect model (glmmTMB in R)) for paternity (log) by non-sibling (n= 7) and sibling fathers (n= 7) within each of the trios. Estimates are provided with standard error (SE), confidence intervals (CI) and degrees of freedom (df1= k -1 and df2= n_tot_ – k, where k is the number of treatment levels and n_tot_ is the total number of observations).*

**Figure S5** *Input parameters for the CASA automated sperm analyses used in this study*
